# Pandemic Influenza (A/H1N1) Vaccine Uptake among French Private General Practitioners: A Cross Sectional Study in 2010

**DOI:** 10.1371/journal.pone.0041837

**Published:** 2012-08-03

**Authors:** Pierre Verger, Rémi Flicoteaux, Michael Schwarzinger, Luis Sagaon-Teyssier, Patrick Peretti-Watel, Odile Launay, Remy Sebbah, Jean-Paul Moatti

**Affiliations:** 1 U912 Sciences Economiques & Sociales de la Santé & Traitement de l’Information Médicale, Institut National de la Santé et de la Recherche Médicale, Marseilles, France; 2 UMR-S912, Aix Marseille Université, Institut Recherche et Développement, Marseilles, France; 3 Observatoire Régional de la Santé Provence-Alpes-Côte d'Azur, Marseilles, France; 4 Département de Biostatistique et d'Information Médicale, Hôpital Saint Louis, Assistance Publique des Hôpitaux de Paris, Paris, France; 5 U738 Action Thématique et Incitative sur Programme-AVENIR, Institut National de la Santé et de la Recherche Médicale, Paris, France; 6 UMR-S738, Université Paris Diderot, Sorbonne Paris Cité, Paris, France; 7 Centre d’Investigations Cliniques BT505, Institut National de la Santé et de la Recherche Médicale, Paris, France; 8 Union régionale des professionnels de santé, médecins libéraux, Provence-Alpes-Côte d'Azur, Marseilles, France; Melbourne School of Population Health, Australia

## Abstract

**Background:**

In July, 2009, French health authorities, like those in many other countries, decided to embark on a mass vaccination campaign against the pandemic A(H1N1) influenza. Private general practitioners (GPs) were not involved in this campaign. We studied GPs’ pandemic vaccine (pvaccine) uptake, quantified the relative contribution of its potential explanatory factors and studied whether their own vaccination choice was correlated with their recommendations to patients about pvaccination.

**Methodology/Principal Findings:**

In this cross-sectional telephone survey, professional investigators interviewed an existing panel of randomly selected private GPs (N = 1431; response rate at inclusion in the panel: 36.8%; participation rate in the survey: 100%). The main outcome variable was GPs’ own pvaccine uptake. We used an averaging multi-model approach to quantify the relative contribution of factors associated with their vaccination. The pvaccine uptake rate was 61% (95%CI = 58.3–63.3). Four independent factors contributed the most to this rate (partial Nagelkerke’s R^2^): history of previous vaccination against seasonal influenza (14.5%), perception of risks and efficacy of the pvaccine (10.8%), opinions regarding the organization of the vaccination campaign (7.1%), and perception of the pandemic's severity (5.2%). Overall, 71.3% (95%CI = 69.0–73.6) of the participants recommended pvaccination to young adults at risk and 40.1% (95%CI = 37.6–42.7) to other young adults. GPs’ own pvaccination was strongly predictive of their recommendation to both young adults at risk (OR = 9.6; 95%CI = 7.2–12.6) and those not at risk (OR = 8.5; 95%CI = 6.4–11.4).

**Conclusions/Significance:**

These results suggest that around 60% of French private GPs followed French authorities’ recommendations about vaccination of health care professionals against the A(H1N1) influenza. They pinpoint priority levers for improving preparedness for future influenza pandemics. Besides encouraging GPs' own uptake of regular vaccination against seasonal influenza, providing GPs with clear information about the risks and efficacy of any new pvaccine and involving them in the organization of any future vaccine campaign may improve their pvaccine uptake.

## Introduction

In March, 2009, a new influenza virus, A(H1N1), appeared in Mexico and rapidly spread to several countries [1]. The first French cases were identified on May 1, in travelers returning from Mexico. On June 11, the World Health Organization (WHO) formally confirmed the pandemic and officially declared a phase 6 alert (the highest) [2]. In July, French health authorities, like those in many other countries, decided to follow the WHO recommendation [2] and embark on a mass vaccination campaign against the pandemic influenza. French authorities organized this campaign in dedicated centers and did not involve private general practitioners (GPs), who normally deliver most vaccinations against seasonal influenza. The reasons for this decision were the multidose presentation of the vaccine and to facilitate monitoring of pandemic vaccination (pvaccination) coverage. Authorities also expected that GPs would be rapidly overwhelmed in caring for the infected patients.

In October, 2009, pvaccination was proposed first to health care workers (HCWs) [3], because they were at high risk of themselves contracting A(H1N1) influenza, might transmit it to patients and medical staff, and were essential participants in combatting the pandemic. This vaccination required HCWs to come to the dedicated centers for pvaccination, which was not available for them to purchase privately and administer themselves until the end of January 2012. Influenza vaccination and then pvaccination were recommended. Moreover, HCWs are role models who play a crucial role in educating their patients about influenza vaccination [4], most especially primary care physicians, regularly consulted by the population, particularly during epidemics [5]. GPs in private practice are the linchpin of primary care in the French health care system [6].

Physicians’ willingness to be vaccinated against pandemic influenza during the prepandemic period varied widely between countries and between types of practice within countries (from ≤20% to ≥80% according to published studies) [7]–[16]. Before the pandemic, 61.7% of GPs in France stated that they were willing to be vaccinated against A(H1N1) and 71% reported that they had been vaccinated against seasonal influenza during each of the three previous years [3]. However, previous studies suggest that the words of HCWs do not always translate into deeds, that is, in this situation, into vaccine uptake [7], [17]. Pandemic vaccine uptake in HCWs has not been extensively studied. In particular, two recent reviews of studies of pvaccination and its determinants in various population groups did not mention studies of GPs’ pvaccine uptake [7], [8]. Most published studies show rather low rates, around or below 20–25% [7], [18]. There is good evidence that past vaccination against influenza is a predictor of A/H1N1 vaccination uptake and that the degree of threat during the 2009 pandemic and perceptions of vaccination (its efficacy, safety and side effects) were associated with uptake rates. People relying on unofficial sources of information have been shown to be less likely to be vaccinated that people relying on official health sources [7]. Controversies about the safety and efficacy of the 2009 pvaccine might have modified French GPs’ decisions, especially given that the pvaccine used in France included an adjuvant [19], [20]. Moreover, the initial decision of French public health authorities to refuse direct involvement by GPs in the A(H1N1) influenza pvaccination campaign might have affected GPs’ personal decisions to be vaccinated, including by making vaccination more inconvenient [19].

At the end of 2010 we conducted a cross-sectional telephone survey to study GPs’ perceptions and behaviors regarding the pvaccination campaign. The aims of the study were to: 1) document retrospectively their pvaccine uptake; 2) quantify the relative contribution of explanatory factors of GPs’ pvaccination with a model-averaging approach; 3) study whether their own vaccination choice was correlated with their recommendations to patients about pvaccination. These aims were achieved.

## Methods

### Sampling

In 2008, around 58,000 GPs (31.6% of them women) were in private practice in France and accounted for 56% of all GPs registered in the French Ministry of Health database; the other GPs are salaried employees of health care facilities or are not involved in health care delivery (e.g., work in administration, research, health insurance, or screening programs) and were thus not the target of our panel [21]. The survey was nested in an existing national panel of French private GPs designed to collect data regularly about their activity and practices. The panel began in June 2010: 5,170 GPs were selected by random sampling from the Ministry of Health's exhaustive database of health professionals in France, ADELI (“Automatisation DEs LIstes”). Sampling was stratified for location of the general practice (urban, suburban, or rural areas), gender, age (<49, 49–56, >56), and annual workload, defined by number of office consultations and house calls (<2849, 2849–5494, >5494) in 2008 (information was obtained for each GP from the exhaustive reimbursement database of the General Health Insurance Fund). We stratified for the latter variable because workload varies substantially between GPs and may influence their decision to participate in the panel. To limit a selection bias that might have resulted from particular opinions/attitudes, the specific topics to be studied were not mentioned to GPs before they were asked to consent to participate in the panel.

### Ethics Statement

GPs who agreed to participate in the panel sent back a signed written consent to our team. The National Data Protection Authority (Commission Nationale Informatique et Libertés), responsible for ethical issues and protection of individual data in France, approved the panel and its procedures.

### Procedure and Questionnaire

The survey about pvaccination took place during the last 2 months of 2010. Professional investigators contacted the panel members and interviewed them with computer-assisted telephone interview (CATI) software, using an ad hoc uniform questionnaire developed on the basis of a literature review and discussions with experts and pilot-tested for clarity, length, and face validity among 50 GPs. The questionnaire collected information about their demographic and professional characteristics and their personal history of vaccination against seasonal influenza in 2007, 2008, and 2009 (Table 1). Respondents were asked if they had agreed, back in July, 2009, with the decision to vaccinate HCWs in priority and whether this opinion had changed in the meanwhile; if and when they had been vaccinated against A(H1N1) pandemic influenza; and if they had recommended the pvaccination (yes/no) to young adults with and without known risk factors of severe flu (pregnancy, chronic diseases such as diabetes mellitus, asthma, etc. [22]). We focused on young adults because of the high proportion of hospitalizations and deaths due to the pandemic influenza in this population group, compared with seasonal influenza [23], [24]. The physicians were also asked about their sources of information about the 2009 A/H1N1 pandemic and the vaccine, their trust in the public health authorities to manage the pandemic, and their opinions of the pandemic's severity and of the efficacy and adverse effects of the pvaccine. We crossed the answers to the questions about vaccine efficacy and about its adverse effects to construct a categorical variable evaluating GPs’ perception of the risks and efficacy of pvaccination (Table 2). Participants were asked whether any of their patients had been hospitalized or had died because of A/H1N1 pandemic influenza. They were also asked about their perception of the pandemic's severity (high/low). We crossed the answers to these two closely correlated (p = 0.004) variables to construct a new categorical variable as a proxy for GPs’ perception of the pandemic threat, which took into account both their clinical experience of pandemic severity and their more general perception of the pandemic (Table 2). In addition, we asked GPs their opinion of the organization of the mass vaccination campaign in dedicated centers.

**Table 1:** Social, demographic, and professional characteristics of GPs according to their vaccination status for A/H1N1 flu (French nationwide panel of general practitioners, weighted data, N = 1431).

	%	% vaccinatedGPs (N = 868.7*)	% unvaccinatedGPs (N = 560.0* ^±^)	p value†
**Gender**				
*Male*	72.7	74.3	70.4	0.10
*Female*	27.3	25.7	29.6	
**Age (years)**				
*<49*	30.9	32.6	28.1	0.12
*49–56*	36.5	34.8	39.4	
*>56 years*	32.6	32.7	32.5	
**Place of practice**				
*Rural*	20.7	21.5	19.6	0.05
*Suburban*	18.0	19.6	15.5	
*Urban*	61.3	58.9	64.9	
**Number of office visits and house calls in 2008**				
*<2849*	22.0	19.2	26.7	0.00
*Between 2849 and 5494*	52.9	53.7	51.7	
*>5494*	25.0	27.1	21.7	
**Practice**				
*Group*	53.5	58.9	45.3	<10^−4^
*Solo*	46.5	41.1	54.7	
**Occasional practice of alternative medicine**				
*No*	86.4	89.7	81.2	<10^−4^
*Yes*	13.6	10.3	18.8	
**History of seasonal flu vaccination during the 2007–2009 winters**				
*0*	18.8	5.6	39.1	<10^−4^
*1–2*	9.7	8.5	11.7	
*3*	71.5	86.0	49.2	
**Number of CME courses in 2009** ‡				
*0*	47.3	40.9	57.0	<10^−4^
*≥1*	52.7	59.1	43.0	

* Weighted numbers;

† Rao-Scott Chi^2^ test;

‡ Number of half-day CME courses on infectious diseases and vaccination.

**Table 2:** Opinions and attitudes of GPs according to their vaccination status for A/H1N1 flu (French nationwide panel of general practitioners, weighted data, N = 1431).

	%	% vaccinated GPs (N = 868.7*)	% unvaccinated GPs (N = 560.0*)	p value†
**Information sources about the pandemic**				
**Mass media**				
*No*	80.1	83.8	74.4	<10^−4^
*Yes*	19.9	16.2	25.6	
**« DGS urgent »** ‡				
*No*	64.0	58.7	72.2	<10^−4^
*Yes*	36.0	41.3	27.9	
**Internet sites in French**				0.50
*No*	28.4	27.7	29.3	
*Yes*	71.6	72.3	70.7	
**Internet sites in English**				0.29
*No*	79.2	80.1	77.7	
*Yes*	20.8	19.9	22.3	
**Medical journals**				
*No*	17.3	18.0	15.9	0.32
*Yes*	82.7	82.0	84.1	
**Perception of pandemic vaccine risks/efficacy**				
*Fear of side effects and doubts about vaccine efficacy*	24.8	13.7	42.2	<10^−4^
*Fear of side effects only*	7.8	4.4	13.2	
*Doubts about vaccine efficacy only*	20.5	20.8	20.1	
*No fear or doubts (favorable)*	46.9	61.1	24.5	
**Perception of pandemic severity**				
*Low, no patients hospitalized*	38.0	28.6	52,9	<10^−4^
*High, no patients hospitalized*	31.8	35.3	26.1	
*Low, patients hospitalized*	13.9	14.4	13.1	
*High, patients hospitalized*	16.3	21.8	7.9	
**Opinions of the health authorities' decisions**				
**Agreement with mass vaccination in centers**				
*Totally disagrees*	53.0	41.8	70.1	<10^−4^
*Partially disagrees to entirely agrees*	47.0	58.2	29.9	
**Lack of trust in public authorities to manage the pandemic**				
*No*	42.4	49.5	31.0	<10^−4^
*Yes*	57.6	50.5	69.0	

* Weighted numbers;

† Rao-Scott Chi^2^ test;

‡ Ministry of Health service providing timely information to physicians during public health emergencies through e-mails.

### Statistical Analysis

Due pto the panel participants' characteristics (see the “results” section), we weighted the data to match the sample more closely to the national French GP population for age, gender, and 2008 workload [25]. The remainder of the paper presents the weighted data, but analyses without weighting produced very similar results.

To take the sample stratification and weights into account we used Rao-Scott Chi-2 tests to examine the univariate associations between the dependent variable, GP pvaccine uptake, and the explanatory variables (Table 2). Only variables significant at p<0.15 in the univariate analysis were included in the multivariate logistic regression models, which were adjusted for age, gender, size of the place of practice, 2008 workload, and type of practice (solo/group). We used a multi-model averaging approach based on the Akaike information criteria (AIC) both to take into account the uncertainty linked to the process of selecting a final model with standard regression procedures and to rank the explanatory variables according to their relative importance. This approach estimates all the possible models, given the explanatory variables introduced, and computes the final model as the weighted average of all the parameters and standard errors from all possible models [26]. We used partial Nagelkerke’s R squares to quantify the partial contributions of each explanatory variable to the GPs’ pvaccine uptake [27]. Nagelkerke's R^2^ is a generalization to binary dependent variables (i.e., logit or probit) of the coefficient of determination (R^2^) used for continuous dependent variables (in traditional linear regression). It compares the likelihood of an empty model (with only an intercept) with the likelihood of the model with explanatory variables. This comparison is interpreted as the proportion of the variation explained by the specified model. We used relative importance weights (values between 0 and 1) to classify the explanatory factors according to the weight of the evidence supporting the presence of an actual relationship with the dependent variable [28] with the following classification [29]: [0–0.5[ = no evidence; [0.5–0.75[ = weak evidence; [0.75–0.90[ = positive evidence; [0.95–0.99[ = strong evidence; [0.99–1 [ = very strong evidence.

We also conducted two multivariate logistic regressions adjusted for age, gender, size of practice area, and 2008 workload to test the association between GPs’ recommendations about pvaccination to young adults both at risk and not at risk (dependent variables) and their own pvaccination (used here as an explanatory variable).

## Results

Of the 5170 private GPs initially selected, 4111 (79.5%) could be contacted after a maximum of 10 attempts; 223 were not eligible, as 112 had already retired or planned to within the next year, 39 planned to move their practice within the next year, and 72 practiced exclusively alternative medicine (e.g., homeopathy or acupuncture). Finally, 1431/3888 eligible and contacted GPs (36.8%) agreed in writing to participate in the panel, i.e., to provide regular data on their professional activity and respond to 5 consecutive surveys during a 30-month period. The GPs who refused to take part did not differ from participants according to practice location, but were more frequently male (p = 0.02), older (p<10^−3^), and had a higher workload in 2008 (p<10^−3^). Lack of time (46.2%) and lack of interest in the panel (15.6%) were the reasons given most frequently for refusal.

All GPs who initially joined the panel participated in this cross-sectional survey: 72.7% were male, 30.9% younger than 49 years, and 36.5% aged 49–56 years; 53.5% were in group practices; 52.9% had 2849–5494 individual patient visits in 2008; 81.2% had been vaccinated against seasonal influenza at least once between 2007 and 2009, and 71.5% in all three of those years (Table 1).

Overall, 61% (95%CI = 58.3–63.3) reported that they had been vaccinated against A/H1N1, although 67.0% had agreed in July 2009 with the authorities’ decision to vaccinate HCWs in priority. Only 23.6% changed their mind about pvaccination during the pandemic, 15.8% becoming negative and 7.8% positive. According to 51.9% of the participants the severity of the pandemic was low; 32.7% thought the side effects of the pvaccine were a matter of serious concern, and 45.3% that there was a lack of reliable efficacy data; 57.6% did not trust the health authorities' ability to manage the pandemic. These percentages were significantly lower among GPs who had been vaccinated against seasonal influenza each year in 2007–2009 than in GPs not vaccinated at all (respectively 47.7%/62.4%, 25.2%/51.3%, 40.6%/57.0% and 54.8%/64.8%; all p<10^−3^).

Univariate analyses showed no association between GPs’ pvaccination and their gender or age. However, compared with unvaccinated GPs, those who were vaccinated (Tables 1 & 2) were in solo practices less often; practiced some alternative medicine less often; reported more frequently regular personal vaccination against seasonal flu and a continuing medical education (CME) courses (at least one half day) about infectious diseases and vaccination within the past year; perceived the risks of the pvaccination to be lower and the efficacy higher (Table 2); consulted official medical sites (e.g., “DGS urgent”) more often and the mass media less often for pandemic information; were less likely to consider the pandemic severity to be low; more frequently had patients hospitalized for A/H1N1 influenza; were more favorable to organization of the vaccination campaign in dedicated centers; and expressed more trust in public health authorities. The averaging model confirmed most of these associations (Tables 3 & 4). In particular, 4 factors strongly influenced GPs' behavior towards pvaccination (partial Nalgelkerke’s R^2^): history of seasonal influenza vaccination (14.5%), perception of the risks/efficacy of the pvaccine (10.8%), opinion about the organization of the pvaccination campaign (7.1%), and perception of pandemic severity (5.2%): total Nagelkerke’s R^2^: 46.8% (Table 4).

**Table 3:** Factors associated with personal vaccination of GPs against A/H1N1 flu (French nationwide panel of general practitioners, multi-model averaging, weighted data, N = 1429).*

	Odds	95% confidence interval
	Ratio	
**Social, demographic, and professional characteristics**		
Type of practice: *group (reference: solo)*	1.47	1.11–1.96
Occasional practice of alternative medicine *(ref: no)*	0.86	0.58–1.28
Seasonal flu vaccination during the last 3 winters† *(ref: zero)*		
* 1 – 2*	4.05	2.36–6.95
* 3*	8.63	5.81–12.82‡
CME sessions# *(ref: zero)*	1.70	1.29–2.23
**Opinions and attitudes during the pandemic**		
**Information sources on the pandemic**		
Mass media *(ref: no)*	0.68	0.48–0.94
DGS urgent§ *(ref: no)*	1.49	1.11–2.00
**Perception of pandemic vaccine risks/efficacy** *(ref: no fear or doubts)*		
* Doubts about efficacy*	0.49	0.34–0.69
* Fear of side effects*	0.20	0.12–0.33
* Doubts about efficacy and fear of side effects*	0.21	0.15–0.29
**Pandemic severity perception** *(ref: low severity, no patients hospitalized)*		
* High severity and no hospitalized patients*	1.86	1.35–2.58
* Low severity and hospitalized patients*	2.23	1.46–3.39
* High severity and hospitalized patients*	4.00	2.54–6.28
**Opinions about health authorities' decisions**		
Partially disagree to entirely agree with the organization of the vaccination campaign in centers *(reference: totally disagree)*	3.08	2.32–4.08
Lack of trust in public authorities to manage the pandemic *(reference: do trust authorities)*	0.84	0.63–1.12

* Model adjusted for gender, age, type of place of practice, 2008 workload, and solo or group practice (only the latter variable is significant);

† Including 2009–2010;

‡ Significant dose-effect relation (p<0.001);

# On infectious diseases and vaccination;

§ Ministry of Health service distributing timely information to physicians during public health emergencies through e-mails.

**Table 4:** Relative contribution and importance of the explanatory factors associated with personal vaccination of GPs against A/H1N1 flu (French nationwide panel of general practitioners, weighted data, N = 1429: multi-model averaging).*

	Partial Nagelkerke's R^2^ (%)	Importance weight	Evidence†	Rank‡
Seasonal flu vaccination history	14.54	1.00	Very strong	1
Perception of pandemic vaccine risks/efficacy	10.84	1.00	Very strong	2
Opinion about the organization of the vaccination campaign in dedicated centers	7.13	1.00	Very strong	3
Perception of the pandemic's severity	5.24	1.00	Very strong	4
CME on infectious diseases and vaccination in the past year	1.70	1.00	Very strong	5
"DGS urgent" was a source of information about pvaccination	0.84	0.93	Positive	6
Mass media were a source of information about vaccination	0.62	0.91	Positive	7
Lack of trust in public health authorities	0.16	0.55	Weak	8
Occasional practice of alternative medicine	0.07	0.29	None	9

* Total Nagelkerke’s R^2^ : 46,77%.

† According to the value of the importance weights [28], [29]:

• [0–0.5[: no evidence.

• [0.5–0.75[: weak evidence.

• [0.75–0.90[: positive evidence.

• [0.95–0.99[: strong evidence.

• [0.99–1 [: very strong evidence.

‡ Based on importance weight.

More than two thirds of the respondents (71.3%; 95%CI = 69.0–73.6) reported recommending pvaccination to young adults at risk of severe flu in their practice, and 40.1% (95%CI = 37.6–42.7) to other young adults. The multivariate logistic regression models adjusted for the stratification variables showed that GPs’ personal uptake of pvaccine was strongly associated with their recommendation of the pvaccination to young adults, both those at risk (OR = 9.6; 95%CI = 7.2–12.6) and those not at risk (OR = 8.5; 95%CI = 6.4–11.4).

## Discussion

To our knowledge, this study is the first to focus on the behaviors of GPs in private practice – rather than on their expressed willingness to be vaccinated against the pandemic A(H1N1) influenza and to recommend this vaccination to patients. French GPs’ pvaccination uptake rate was lower than the vaccine uptake found among Dutch GPs (85%) in a mail survey [30]. Those authors attribute this high rate in part to a campaign by the Dutch government and the Dutch College of General Practitioners that strongly and repeatedly urged HCWs to be vaccinated against A/H1N1. We are not aware of other studies of pvaccination uptake in GPs [7], [8]. However, our result was high compared to the rates observed in most studies of hospital physicians in Western countries (<15.0% to ≤50.0%) and elsewhere [7], [8], [18], [31]–[34], even those based, as ours was, on questionnaires, a method that might overestimate pvaccination uptake [8]. The relatively high rate among French GPs may be explained by their first-line role in the care of seasonal and pandemic influenza and their consequently high risk of exposure to the corresponding viruses [35]. Under these circumstances, most French GPs wanted to avoid being unable to work during an epidemic when patient demand is highest [5].

French GPs’ pvaccination uptake was close to the percentage of French GPs who stated they were willing to accept the vaccination (61.7%) in a previous survey during the prepandemic period [3]. These positive words about the pvaccination appeared to be transformed into deeds – vaccination – despite the controversy surrounding it in France [19], [20], especially the pandemic severity and the safety of the vaccine (choice of a pvaccine including an adjuvant, risk of Guillain-Barre syndrome [36]). Most GPs (67.0%) said that they had agreed in July 2009 with the statement that HCWs should be vaccinated in priority against A/H1N1, and this opinion was confirmed by an even greater majority (>75%) after the pandemic. Actual uptake of the pvaccine among hospital HCWs in other countries was lower than the stated intentions during the prepandemic period [34].

Our results about the determinants of pvaccination uptake are consistent with previous results for HCWs and with population studies that suggest the role of past vaccination against influenza, degree of perceived threat during the 2009 pandemic, perceptions of the benefits and risks associated with pvaccination, and sources of information about the pandemic (Bish et al. 2011). To our knowledge however, our study is the first to attempt to quantify the relative contribution of these factors, including a factor concerning the pvaccination campaign organization [7], [8], [30]. Such information could be useful to help prioritize and design the components of programs aiming at improving pandemic preparedness. GPs' history of regular influenza vaccination was by far the most important factor independently associated with their pvaccination [3], [33], [34]). Well-run seasonal influenza vaccination campaigns thus appear essential to prepare the ground for the next pandemic, especially in the face of inaccurate perceptions about its potential risks [34].

Such campaigns, however, while necessary, will apparently not be sufficient, for a considerable proportion of the GPs regularly vaccinated against seasonal influenza reported doubts about the efficacy of the pvaccine and fears of its side effects. These doubts may have been due to concerns about using new vaccines during a pandemic, probably different from concerns about established products in non-crisis situations [37]. Our results suggest that GPs’ decisions to be vaccinated against A(H1N1) were based in part on their assessment and comparison of the perceived risk and the perceived benefits associated with pvaccine uptake (see Table 3, the variable for the perception of pandemic vaccine risks and efficacy), as also found among the general population [38]. During this process GPs appeared to give more weight to the vaccine's safety than to its efficacy in making their decisions. Our results also suggest that they applied the same kind of reasoning for their patients, taking level of vulnerability to A(H1N1) influenza into account in advising young adults about the pvaccination.

Some dissonance between GPs’ behavior and their perceptions of the pvaccine nonetheless appeared (Table 2). A quarter of the unvaccinated GPs (A/H1N1) were quite favorable to the pvaccine: as observed elsewhere [34], they probably did not perceive themselves as at risk of contracting this flu or developing serious consequences related to it. At the same time, nearly one third of those vaccinated expressed doubts about its efficacy.

Although the ranking of explanatory factors might have been different had the pandemic been more severe [39], our results add further evidence to the need to design effective strategies to inform GPs (and all HCWs) about the risks and efficacy of a new pvaccine and to avoid or correct false opinions about it. These strategies should consider multiple channels of information, given that GPs use various sources to obtain their information. The inverse relation between GPs’ pvaccination and their use of mass media for pandemic information (Table 3) suggests that the controversy about the pvaccine safety in the mass media induced or reinforced GPs’ inaccurate opinions and their doubts about the new vaccine [40].

GPs’ personal acceptance of pvaccination appeared strongly predictive of their recommendation of it to the young adults in their practice – at risk or not. This correlation is not surprising, as it exists for seasonal vaccinations [4]. Such a correlation suggests that ensuring high pvaccination uptake in GPs might be one lever for improving pvaccination uptake in the general population, especially as GPs’ positive advice significantly increased acceptability of the pvaccination among adults [19]. However, French health authorities’ decision to conduct the pvaccination campaign in dedicated centers implied that GPs were not involved in its implementation, although they usually play a central role in vaccination education and follow-up of their patients [41]. This probably produced a supplementary barrier to acceptance of pvaccination: uptake in the French general adult population was between 3% and 8% depending on age [20] and 22.7% in pregnant women. Indeed our results suggest that GPs’ disagreement with this decision was the third most important obstacle to their personal uptake of pvaccination (Table 4).

### Strengths and Limitations of the Study

The participation rate in this national French private GP panel was 36.8%, a relatively high rate for panels of physicians requiring participation in repeated surveys (see for example [42]: response rate = 19%). Participants in the panel differed from nonparticipants for gender, age, and 2008 workload, but weighting our data by these variables did not affect our results; this is reassuring regarding the magnitude of a potential selection bias. The survey on pvaccination was cross-sectional and retrospective. Therefore, the observed links should be interpreted with caution, as recall bias and a posteriori rationalization may have affected GPs’ responses about their attitudes and actions [43]. However, these would probably have had a much stronger effect had the study been carried out in the midst of the controversy, rather than several months later when the media had left the controversy behind. Using the multi-model averaging approach allowed us to draw inferences from a set of plausible models rather than from a single model [44]. Given the set of potential explanatory variables, this technique accounts for all the possible configurations and summarizes all this information in a final composite model. In doing so, one part of the uncertainty linked to the process of selecting the final model is controlled. A weakness of this approach, however, is that uncertainty is controlled only partially: the multi-model inference is made under the assumption that the observed variables are sufficient to explain a given phenomenon and do not account for unobserved heterogeneity or omitted variables [45], a limitation it shares with other modeling approaches.

### Conclusions

Analysis and quantification of the relative contribution of the factors associated with private GPs’ pvaccination allowed us to pinpoint priority components of preparedness that can be improved for future influenza pandemics. In particular, the results suggest that efforts should be devoted to encouraging regular uptake of seasonal influenza vaccination among GPs, as well as providing them with clear information on the risks and efficacy of a new pvaccine and to putting in place an organizational framework for future mass vaccination campaigns that would allow their direct involvement in the vaccination process at the population level.
